# Emerging venetoclax plus hypomethylating agent-based triplet regimens in newly diagnosed acute myeloid leukemia: updates from the 2025 ASH Annual Meeting

**DOI:** 10.1186/s40164-026-00810-3

**Published:** 2026-07-27

**Authors:** Haibo Yu, Ting Shi, Hong-Hu Zhu

**Affiliations:** 1 Department of Hematology, Beijing Chao-Yang Hospital, Capital Medical University, Beijing, 100020 China; 2 Chinese Institutes for Medical Research, Beijing, 100069 China; 3 Institute for Cancer, Chinese Institutes for Medical Research (CIMR), Beijing, 100069 China

**Keywords:** Newly diagnosed, Acute myeloid leukemia, Venetoclax, Hypomethylating agents, Triplet regimen

## Abstract

Venetoclax (VEN) combined with a hypomethylating agent (HMA) has become a standard-of-care frontline therapy for unfit patients with newly diagnosed acute myeloid leukemia (ND AML). The VIALE-A trial established the clinical benefit of VEN plus azacitidine (AZA), with a composite complete remission rate (CRc) of 66.4% and a median overall survival (OS) of 14.7 months. Nevertheless, remission durability remains heterogeneous, and the regimen is non-curative for many patients. Improving remission rates and long-term survival therefore remains an urgent unmet clinical need. VEN/HMA-based triplet regimens incorporating targeted agents are emerging as promising strategies in ND AML, particularly FLT3 inhibitor–based combinations reported at the 2025 American Society of Hematology (ASH) Annual Meeting. Notably, longer-term follow-up data from a study of gilteritinib plus VEN/AZA in patients with ND FLT3-mutated AML showed a median OS of 29.7 months. Additional VEN/HMA-based strategies, including combinations with menin inhibitors and other novel agents, are also being investigated. Here, we provide an overview of the latest clinical updates on VEN/HMA-based triplet regimens in ND AML presented at the 2025 ASH Annual Meeting.

**To the Editor,**

While venetoclax (VEN) plus a hypomethylating agent (HMA) is a standard frontline therapy for unfit patients with newly diagnosed acute myeloid leukemia (ND AML), limited efficacy in specific genetic subgroups and drug resistance remain major challenges. Recent trials evaluated VEN/HMA-based triplets incorporating a third targeted agent to deepen responses and improve long-term outcomes. This report summarizes the latest developments in novel VEN/HMA-based triplet regimens for ND AML presented at the 2025 American Society of Hematology Annual Meeting (Table 1).

**Table 1:** Efficacy and safety of venetoclax plus hypomethylating agent-based triplet regimens for newly diagnosed acute myeloid leukemia presented at the 2025 American Society of Hematology Annual Meeting

Author	Study design	Regimen	VEN schedule	Disease subtype	No. of patients	Median age (range)	Patient characteristics	Median follow-up	Response	Bridged to HSCT	Adverse events	Survival
Azevedo [1]	Phase II	Gilteritinib/VEN/AZA	C1: VEN 400 mg D1–28; C2–C24: VEN 400 mg D1–7	ND, unfit FLT3m AML	30	71 (18–86)	FLT3-ITDm: 73%; FLT3-TKDm: 27%	41.5 months	Previously reported: CRc, 96%; MRD neg, 93%	47%	Grade ≥3 AEs: infection, 63%; febrile neutropenia, 40%; sepsis, 17%	Median RFS: 23.4 months; 3-year RFS: 43%; Median OS: 29.7 months; 3-year OS: 46%
Altman [2]	Phase I/II	Gilteritinib/VEN/AZA	VEN 200 or 400 mg D1–28	ND, unfit FLT3m AML	44	VEN 200 mg vs. 400 mg:73 vs. 77	VEN 200 mg vs. 400 mg: FLT3-ITDm, 65% vs. 83%; FLT3-TKDm, 35% vs. 13%	VEN 200 mg vs. 400 mg: 14 vs. 12 months	VEN 200 mg vs. 400 mg: CRc, 90% vs. 91%; CR, 70% vs. 65%; MRD neg, 85% vs. 57%	NA	VEN 200 mg vs. 400 mg: DLTs, 0% vs. 8.3%; TEAEs, 85% vs. 79%	VEN 200 mg vs. 400 mg: 12-month OS, 64% vs. 77%
Azevedo [3]	Phase I/II	Gilteritinib/VEN/ASTX727	C1: VEN 400 mg D1–28; from C2: VEN D1–14	ND/RR, unfit FLT3m AML, high-risk MDS/MPN	39 (ND, 15; RR, 24)	ND: 73; RR: 67	FLT3-ITDm: 69%; FLT3-TKDm: 21%	ND: NA RR: 25.9 months	ND: CRc, 80%; CR, 67%; MRD neg, 75% RR: CRc, 46%; CR, 17%; MRD neg, 38%	ND: 33%	Grade ≥3 TEAEs: febrile neutropenia, 31%; pneumonia, 31%; sepsis, 31%; 8-week mortality: 8%	ND: NA; RR: Median OS, 7.7 months
Arora [4]	Phase II	Gilteritinib/VEN/AZA	NA	ND, FLT3-wt AML	15	71 (52–86)	ELN 2022 risk: adverse, 100%; TP53m: 60%	7.6 months	CRc: 53%; MRD neg: 71%	27%	Grade ≥3 AEs: infection, 40%; febrile neutropenia, 33%; hypertension, 20%	Median RFS: NR; Median OS: NR; Alive at last follow-up: 60%
Jen [5]	Phase II	Revumenib/VEN/ASTX727	VEN ramp-up to 400 mg D1–14	ND, unfit NPM1m, KMT2Ar or NUP98r AML or MPAL	17	68 (60–83)	ELN 2022 risk: favorable, 53%; intermediate, 12%; adverse, 35%; Molecular subgroup: NPM1m, 65%; KMT2Ar, 35%	6 months	ORR: 94%; CR: 88%; MRD neg: 100%	29%	Grade 3 infections: 53%; DS: 24%; QTc prolongation: 47%	Median EFS: NR; Median OS: NR
Roboz [6]	Phase Ib	Ziftomenib/VEN/AZA	Standard dosing	ND, NPM1m AML	39	75 (53–93)	FLT3m: 31%; IDHm: 23%	16.3 weeks	ORR: 94%; CRc: 84%; CR: 58%; MRD neg: 54%	NA	Grade ≥3 TEAEs: 74% (neutropenia, 31%; thrombocytopenia, 23%); Grade ≥3 ziftomenib-related AEs: 36% (neutropenia, 15%; thrombocytopenia, 13%; QTc prolongation, 3%)	Median DOR: NR; Median OS: NR
Watts [7]	Phase Ib	Pegargiminase/VEN/AZA	C1: VEN D1–28; from C2: VEN D1–21, with reductions allowed	ND, unfit AML	25	71.9 (58.1–86.4)	ELN risk: adverse, 100%; TP53m or CMK: 40%; blast-phase MPN: 20%	16.9 months	CRc: 91.3%; CR: 69.6%; MRD neg: 52.4%	20%	No DLTs; 30-day mortality: 8%	Median DOR: 10.4 months; Median OS: 19.1 months
Lachowiez [8]	Phase Ib	Iadademstat/VEN/AZA	VEN 400 mg D1–21	ND AML or MDS/AML	8	70 (64–81)	ELN 2022 risk: intermediate, 12%; adverse, 88%	9 months	ORR: 100%; CR: 88%	NA	No DLTs; Grade ≥3 AEs: febrile neutropenia, 25%; neutropenia, 13%; tumor lysis syndrome, 13%; xerostomia, 13%	6-month OS: 88%
Garciaz [9]	Phase I/II	ICT01/VEN/AZA	Standard dosing	ND, unfit AML	54	75 (51–87)	ELN 2024 risk: favorable, 30%; intermediate, 28%; adverse, 42%	8.5 months	ICT01 10 mg vs. 75 mg: CRc, 84% vs. 69%, *P* = 0.02; CR, 68% vs. 44%, *P* = 0.0002	NA	No DLTs; Grade ≥3 febrile neutropenia: 51%; 30-day mortality: 4%	ICT01 10 mg10mg vs. 75 mg: 9-month OS: 74% vs. 56%
Liu [10]	Prospective	Selinexor/VEN/AZA	VEN ramp-up to 400 mg D1–14	ND AML	53	66 (28–83)	ELN 2022 risk: intermediate, 66%; adverse, 34%	NA	ORR: 90%; CRc: 80%	NA	Neutropenia: 70%; thrombocytopenia: 41%; infections: 30%; asthenia: 30%	NA

AML, acute myeloid leukemia; ND, newly diagnosed; RR, relapsed/refractory; MDS, myelodysplastic syndrome; MPN, myeloproliferative neoplasm; MPAL, mixed-phenotype acute leukemia; unfit, intensive chemotherapy-ineligible; HSCT, hematopoietic stem cell transplantation; FLT3m, FLT3-mutated; FLT3-ITDm, FLT3 internal tandem duplication-mutated; FLT3-TKDm, FLT3 tyrosine kinase domain-mutated; FLT3-wt, FLT3-wild type; TP53m, TP53-mutated; KMT2Ar, KMT2A rearranged; NPM1m, NPM1-mutated; NUP98r, NUP98 rearranged; IDHm, IDH-mutated; CMK, complex or monosomal karyotype; ELN, European LeukemiaNet; CR, complete remission; CRc, composite complete remission; CRi, complete remission with incomplete hematologic recovery; CRh, complete remission with partial hematologic recovery; ORR, overall response rate; MRD neg, measurable residual disease negativity; OS, overall survival; EFS, event-free survival; RFS, relapse-free survival; DOR, duration of response; NR, not reached; NA, not available; VEN, venetoclax; AZA, azacitidine; ASTX727, decitabine/cedazuridine; DLTs, dose-limiting toxicities; AEs, adverse events; TEAEs, treatment-emergent adverse events; QTc, corrected QT interval; DS, differentiation syndrome; C, cycle; D, day

^&^ELN risk classification: The 2022 ELN [11] recommendations stratify AML into favorable-risk, intermediate-risk, and adverse-risk groups based on cytogenetic and molecular features in patients treated with intensive chemotherapy; TP53-mutated AML is categorized as adverse-risk disease. The 2024 ELN [12] recommendations updated the genetic risk classification for AML in the context of less-intensive therapies

^%^MRD negativity definitions: MRD negativity was defined according to the assay and threshold reported in each original study. Unless otherwise specified, MRD negativity refers to multiparameter flow cytometry (MFC)-based MRD negativity. In Roboz et al. [6], local MRD was assessed by next-generation sequencing, reverse transcription quantitative polymerase chain reaction and MFC

*CRc definitions: CRc definitions varied across studies. Altman et al. [2] did not define CRc. Watts et al. [7] and Lachowiez et al. [8] defined CRc as CR + CRi + CRh, whereas the remaining studies reporting CRc defined CRc as CR + CRi

## VEN/HMA + FLT3 inhibitors

Standard VEN plus azacitidine (AZA) showed suboptimal efficacy for unfit patients with FLT3-mutated ND AML. A phase II trial of gilteritinib (GILT) plus VEN/AZA reported a composite complete remission (CRc) rate of 96% and a multiparameter flow cytometry (MFC)-based measurable residual disease (MRD) negativity rate of 93%. After a median follow-up of 41.5 months, the median relapse-free survival (RFS) was 23.4 months and the median overall survival (OS) was 29.7 months, with 3-year RFS and OS rates of 43% and 46%, respectively [1]. However, most patients required dose and treatment duration reductions due to myelosuppression. To optimize dosing, the randomized phase I/II VICEROY trial compared VEN at 200 mg versus 400 mg daily in combination with standard-dose AZA and GILT [2]. Both cohorts achieved high CRc rates (90% vs. 91%), MFC-MRD negativity rates (85% vs. 57%) and 12-month OS rates (64% vs. 77%), leading to the selection of VEN 400 mg as the recommended phase 2 dose. A phase I/II study further assessed the all-oral regimen of ASTX727 (decitabine/cedazuridine), VEN and GILT in ND FLT3-mutated myeloid neoplasms [3]. The CRc rate was 80%, including a complete remission (CR) rate of 67%, and the MFC-MRD negativity rate was 75%. Longer follow-up is needed to validate survival outcomes.

In addition, GILT/VEN/AZA exerted potent activity in high-risk FLT3-wild-type AML, possibly via suppression of MCL-1 to enhance VEN-mediated apoptosis. Arora et al. [4] evaluated this regimen in 15 patients with ELN 2022 adverse-risk FLT3-wild-type AML, reporting a CRc rate of 53% and an MFC-MRD negativity rate of 71%. In this cohort, 60% of patients had TP53 mutations and 47% had therapy-related AML. Among patients with TP53-mutated AML, the CRc rate was 44%. With a median follow-up of 7.6 months, median RFS and OS were not reached.

Overall, GILT/VEN/AZA triplet regimens appeared to improve upon the efficacy of conventional VEN/AZA, while rational dose and schedule optimization might help mitigate myelosuppression.

## VEN/HMA + menin inhibitors

Beyond FLT3 inhibitor–based triplet strategies, emerging evidence supported the rational incorporation of menin inhibitors into the VEN/HMA regimen. The phase II SAVE trial explored ASTX727, VEN and revumenib in ND menin-dependent AML and mixed-phenotype acute leukemia [5]. The regimen yielded an overall response rate (ORR) of 94% (16/17) and a CR rate of 88% (14/16), with MFC-MRD negativity in all responders. All patients with KMT2A-rearranged AML achieved fluorescence in situ hybridization negativity. In patients with NPM1-mutated AML, molecular MRD clearance by next-generation sequencing (NGS) was observed in 44% of cases. At a median follow-up of 6 months, median event-free survival (EFS) and OS were not reached. Ziftomenib plus VEN/AZA was also investigated in 39 patients with ND NPM1-mutated AML [6]. It had an ORR of 94% with a CRc rate of 84%. MRD negativity by NGS, reverse transcription quantitative polymerase chain reaction, or MFC was observed in 54% of patients with CRc. After a median follow-up of 16.3 weeks, median EFS and OS were not reached.

Menin inhibitor/VEN/HMA triplets represented a rational strategy for menin-dependent AML, although longer follow-up was needed to confirm response durability and survival benefit.

### VEN/HMA + other novel agents

Several other VEN/HMA-based triplet strategies targeting broader leukemia vulnerabilities were also presented, including metabolic dependency, epigenetic regulation, immune activation and nuclear export. The arginine-degrading agent pegargiminase plus VEN/AZA produced a high CRc rate of 91.3% and a median OS of 19.1 months in older or unfit patients with ND AML, supporting further investigation of metabolic targeting with VEN/AZA [7].

Iadademstat, a lysine-specific demethylase 1 inhibitor, plus VEN/AZA yielded an ORR of 100% and a CR rate of 88% in 8 patients, with an estimated 6-month OS rate of 88% [8]. The most common grade ≥3 adverse events included febrile neutropenia, tumor lysis syndrome and xerostomia. However, the small sample size and limited follow-up warrant further investigation.

ICT01, an anti-butyrophilin 3A monoclonal antibody that activates γ9δ2 T cells, was evaluated with VEN/AZA in 54 older or unfit patients with ND AML [9]. The ICT01 10 mg cohort showed higher CRc rates (84% vs. 69%), CR rates (68% vs. 44%) and 9-month OS rates (74% vs. 56%) than the 75 mg cohort, with no dose-limiting toxicities reported, suggesting that γ9δ2 T-cell activation may enhance VEN/AZA activity.

The XPO1 inhibitor selinexor plus VEN/AZA also showed preliminary activity, with a CRc rate of 80% and MFC-MRD negativity in 25 of 51 efficacy-evaluable patients [10].

In conclusion, these studies support molecularly guided VEN/HMA-based triplet strategies in ND AML. GILT/VEN/AZA in FLT3-mutated AML achieved a CRc rate of up to 96% and a median OS of 29.7 months, while menin inhibitor–based triplets in NPM1-mutated AML reported CR rates of up to 88%; other triplets also showed substantial CRc rates of approximately 80–90%. These outcomes appear favorable compared with VIALE-A, supporting the rationale for adding third targeted agents to the VEN/HMA backbone. However, myelosuppression and infection remain key concerns. Future prospective trials, including ongoing studies evaluating IDH1 inhibitor–based triplet regimens, will be essential to optimize dosing, refine patient selection and confirm survival benefit.

## Data Availability

No datasets were generated or analysed during the current study.

## Abbreviations

ASH: American Society of Hematology
ASTX727: Decitabine/Cedazuridine
AZA: Azacitidine
CRc: Composite complete remission
CR: Complete remission
EFS: Event-free survival
GILT: Gilteritinib
HMA: Hypomethylating agent
NGS: Next-generation sequencing
MFC: Multiparameter flow cytometry
MRD: Measurable residual disease
ND AML: Newly diagnosed acute myeloid leukemia
Unfit: Intensive chemotherapy-ineligible
ORR: Overall response rate
OS: Overall survival
RFS: Relapse-free survival
VEN: Venetoclax
